# Deficient Mechanical Activation of Anabolic Transcripts and Post-Traumatic Cartilage Degeneration in Matrilin-1 Knockout Mice

**DOI:** 10.1371/journal.pone.0156676

**Published:** 2016-06-07

**Authors:** Yupeng Chen, Jack Cossman, Chathuraka T. Jayasuriya, Xin Li, Yingjie Guan, Vera Fonseca, Kun Yang, Cherie Charbonneau, Hongchuan Yu, Katsuaki Kanbe, Peter Ma, Eric Darling, Qian Chen

**Affiliations:** 1 Bone and Joint Research Center, The First Affiliated Hospital and Frontier Institute of Science and Technology, Xi’an JiaoTong University, Xi’an, 710061, China; 2 Department of Orthopaedics, Rhode Island Hospital and Warren Alpert Medical School of Brown University, Providence, RI, 02903, United States of America; 3 Department of Molecular Pharmacology, Physiology, and Biotechnology, Brown University, Providence, RI, 02912, United States of America; 4 Department of Orthopaedic Surgery, Tokyo Women’s Medical University, Medical Center East, Tokyo, Japan; 5 Department of Biologic and Materials Science, University of Michigan, Ann Arbor, Michigan, 48109, United States of America; 6 Center for Biomedical Engineering, Brown University, Providence, RI, 02912, United States of America; University of Alabama at Birmingham, UNITED STATES

## Abstract

Matrilin-1 (*Matn1*), a cartilage-specific peri-cellular and extracellular matrix (ECM) protein, has been hypothesized to regulate ECM interactions and transmit mechanical signals in cartilage. Since *Matn1* knock-out (*Matn1*^*-/-*^) mice exhibit a normal skeleton, its function *in vivo* is unclear. In this study, we found that the anabolic *Acan* and *Col2a* transcript levels were significantly higher in wildtype (*Matn1*^*+/+*^) mouse cartilage than that of *MATN1*^*-/-*^ mice *in vivo*. However, such difference was not observed between *Matn1*^*+/+*^ and *MATN1*^*-/-*^ chondrocytes cultured under stationary conditions *in vitro*. Cyclic loading significantly stimulated *Acan* and *Col2a* transcript levels in *Matn1*^*+/+*^ but not in *MATN1*^*-/-*^ chondrocytes. This suggests that, while *Matn1*^*+/+*^ chondrocytes increase their anabolic gene expression in response to mechanical loading, the *MATN1*^*-/-*^ chondrocytes fail to do so because of the deficiency in mechanotransduction. We also found that altered elastic modulus of cartilage matrix in *Matn1*^*-/-*^ mice, suggesting the mechanotransduction has changed due to the deficiency of *Matn1*. To understand the impact of such deficiency on joint disease, mechanical loading was altered *in vivo* by destabilization of medial meniscus. While *Matn1*^*+/+*^ mice exhibited superficial fissures and clefts consistent with mechanical damage to the articular joint, *Matn1*^*-/-*^ mice presented more severe cartilage lesions characterized by proteoglycan loss and disorganization of cells and ECM. This suggests that *Matn1* deficiency affects pathogenesis of post-traumatic osteoarthritis by failing to up-regulate anabolic gene expression. This is the first demonstration of *Matn1* function *in vivo*, which suggests its protective role in cartilage degeneration under altered mechanical environment.

## Introduction

Matrilin-1 (Matn1) is a cartilage specific non-collagenous extracellular matrix (ECM) protein [1], which has been implicated in diseases such as adolescent idiopathic scoliosis [2] and relapsing polychondritis [3]. Matn1 is a member of the matrilin family, which consists of four proteins sharing similar structural motifs such as von Willebrand factor A (vWFA) domains, epidermal growth factor (EGF)-like domains, and coiled-coil domains [4–6]. Matrilins form homo- or hetero-oligomers through assembly of C-terminal coiled-coil structures [5] and are known to have a bridging role in the ECM by connecting matrix components to form macromolecular networks. Matn1 interacts with type II collagen by coating the surface of collagen filbrils [7]. It also interacts with aggrecan, and such interaction is stabilized by cross-linking [8]. It has also been shown to associate with collagens type VI and IX and biglycan, thereby connecting these molecules to the aggrecan and type II collagen networks [9,10]. Indeed, a major phenotype of *Matn1*^*-/-*^ mice is alteration of type II collagen fibrillogenesis and fibril organization [11]. The collagen fibril diameters are significantly increased, which leads to dense collagenous matrix structure in cartilage [2,11]. However, since skeletal development and growth plate morphology is apparently normal in *MATN1-/-* mice [11], the function of Matn1 *in vivo* in unclear.

Matn1 has also been proposed to play a role in mechanotransduction, since it is abundantly distributed in the pericellular matrix that is involved in transduction of mechanical signals to chondrocytes [12]. Mechanical transduction is the process of translating mechanical stimulation into cellular responses. Mechanical stress plays a fundamental role in regulating cellular activities during tissue morphogenesis and homeostasis [13–15]. Previous studies have shown that moderate cyclic loading stimulates chondrocyte functions by increasing the expression of anabolic chondrogenic ECM molecules such as type II collagen and aggrecan [10,12,13,16]. Elimination of matrilin content abolishes mechanical stimulation of chondrocyte proliferation and differentiation, and excessive or reduced Matn1 content decreases the mechanical response of chondrocytes [12]. It is proposed that pericellular Matn1 may affect overall mechanical adaptation of cartilage by regulating mechanical transduction of chondrocytes [12].

Given the function of Matn1 in regulating collagen fibril organization and in chondrocyte adaptability to mechanical loading, we propose the hypothesis that Matn1 deficiency would alter cartilage matrix mechanical property and chondrocyte mechanotransduction in response to mechanical loading. If this hypothesis is true, one would predict that Matn1 deficiency affects pathogenesis of post-traumatic osteoarthritis (PTOA), which is induced by altered mechanical environment in the joint [17–20]. In this study, we tested this hypothesis by characterizing articular cartilage in *Matn1* wild-type (*Matn1*^*+/+*^), heterozygous (*Matn1*^*+/-*^) and knock-out (*Matn1*^*-/-*^) mice molecularly, mechanically and histologically.

## Materials and Methods

### RT-PCR expression analysis

Total RNA was isolated from rib, femoral head, and knee joint cartilage of 1 week old *Matn1*^*+/+*^ and *Matn1*^*-/-*^ mice of both genders and from femoral head cartilage of 3 week-old *Matn1*^*+/+*^, *MATN1*^*-/-*^ and *Matn1*^*+/-*^ mice of both genders using the RNAqueous® Kit (Ambion, Austin, TX, USA) according to the manufacturer's instructions. Gene expression analysis was conducted by real time quantitative PCR (RT-qPCR) with the DNA Engine Opticon® 2 (Bio-Rad, Hercules, CA, USA) using the QuantiTect SYBR Green PCR kit (Qiagen). For RT-qPCR, 0.5 ug of RNA was reverse-transcribed using iScript™ cDNA synthesis kit (Bio-Rad) according to the manufacturer's instructions. The cDNA of each sample was subjected to RT-qPCR using species-specific primer pairs for genes encoding *Matn1*, type II collagen (*Col2a1*), aggrecan (*Acan*), Indian hedgehog (Ihh), type X collagen (Col X), runt-related transcription factor 2 (Runx2), matrix metalloproteinases 13 (MMP13) and a disintegrin and metalloproteinase with thrombospondin motifs 5 (ADAMTS5). Relative transcript levels were calculated using the delta-delta Ct (ΔΔCt) method, normalized to rRNA 18S expression according to the following equation: x = 2-ΔΔCt, in which ΔΔCt = ΔE - ΔC, and ΔE = Ct_exp_-Ct_18_s; ΔC = Ct_ctl_-Ct_18s_. Ct_ctl_ = Ct of control group.

### Mechanical testing of articular cartilage

Atomic force microscopy (AFM) analysis was used to assess the mechanical changes in *Matn1*^*-/-*^(n = 6), *Matn1*^*+/+*^ (n = 4), and *Matn1*^*+/-*^ (n = 5) articular cartilage surface. The femoral heads of 3 week-old mice of both genders were harvested for evaluation purposes. All freshly harvested samples were wrapped in phosphate-buffered saline (PBS)-soaked gauze and stored at 4°C for up to 2 days post-harvest, following previously established procedures [21]. Prior to testing, whole femurs were glued to a low profile 50 mm Petri dish to allow unobstructed access to the femoral head, and submerged in PBS to prevent drying of the tissue. Elastic moduli were quantitatively evaluated using an MFP-3D AFM (Asylum Research, Santa Barbara, CA) as described previously [13]. Briefly, spherically tipped, 5 μm diameter AFM cantilevers (k ~ 7.5 N/m, Novascan Technologies, Inc., Ames, IA) were used for elastic indentation tests. Indentation curves (10 μm/s approach velocity) were sampled at 5 kHz, with a force trigger between 50–300 nN prescribing the point at which the cantilever approach was stopped and then retracted (approximately 0.5–1 μm of indentation). For each testing region, 100 data points were collected, resulting in n = 400–600 indentations for each genotype group included in the study. Indentation curves were fit using a modified Hertz model to extract an elastic modulus for the cartilage surface [21].

### Mechanical response of chondrocytes

Mouse primary chondrocytes were isolated from the rib cage cartilage of 1 week-old *Matn1*^*+/+*^and *Matn1*^*-/-*^ mice of both genders. Cartilage slices were digested enzymatically in 0.2% collagenase type II. Individual cells were grown in Dulbecco's Modified Eagle Medium (DMEM) containing 10% fetal calf serum. Then, *Col2a1* and *Acan* expression were determined via real time RT-PCR.

Mechanical response of chondrocytes was determined in an established 3D chondrocyte culture system [22]. Chondrocytes cultured in 3D collagen scaffoldings form cartilage-like nodules that express specific markers of cartilage, including *Col2a1* and *Acan*. After incubation of chondrocytes in collagen sponges overnight, the sponges were mechanically loaded to induce 5% elongation at 60 cycles/min, 15 min/h by a computer-controlled device (Bio-Stretch; ICCT Technologies, Markham, ON, Canada) for 48 hours. Bio-Stretch exerts a uniaxial stretch with square wave patterns, which induces extension of the collagen sponges at the x-axis and compression at the y-axis. At the indicated mechanical loading duration, chondrocytes were freed from sponges by collagenase digestion and collected for further analysis.

### Microarray analysis

Rib cage cartilages were isolated from 1 week-old *Matn1*^*+/+*^ or *Matn1*^*-/-*^ mice of both genders. Rib cages were treated with 3mg/ml collagenase D (sigma) in DMEM medium supplemented with 2 mM L-glutamine, 0.05 mg/mL penicillin and 0.05 mg/mL streptomycin. After the first 90min treatment with collagenase D, rib cages were washed with Hank’s Balance Salt Solution (HBSS) by pipetting up and down to get rid of soft tissues surrounding ribs. After another 4 hours treatment with collagenase D or until the cartilages were completely digested, ribs were washed with HBSS solution. Chondrocytes in the supernatant were spin down and total RNA was isolated from them using the RNAqueous® Kit (Ambion, Austin, TX, USA) according to the manufacturer's instructions. Total RNA samples were processed for microarray hybridization by the Genomics Core Facility at the Brown University Center for Genomics and Proteomics with GeneChip® Mouse Gene 1.0 ST Arrays (Affymetrix, USA). Biological triplicates were used for each genetic group. RNA gel electrophoresis is performed to examine RNA integrity. Global gene expression pattern with Principal Components Analysis mapping was generated with Partek Genomics suite 6.6 beta (Partek Incorporated, Missouri, USA).

### Destabilization of the Medial Meniscus

After obtaining Rhode Island Hospital Institutional Animal Care and Use Committee approval, we performed a minimally invasive well known procedure called destabilization of the medial meniscus (DMM) to induce arthritis [23]. The procedure was performed on the right knees of *Matn1*^-/-^ (n = 11) and *Matn1*^+/+^ (n = 9) 2 month-old male mice. By transecting the meniscotibial ligament, a resultant displacement of the medial meniscus produced a mechanical instability of the ipsilateral knee. As a result, weight-bearing was focused in a smaller area on the medial aspects of the posterior femur and the central tibia. With unrestricted movement of the joint, an accelerated age-onset osteoarthritis (OA) model was induced by high focal mechanical stresses. Sham surgery was performed in which the ligament was visualized but not transected. Twenty-five mg/kg cefotaxime was injected after surgery to prevent infection, and 0.03mg/kg buprenophine was injected for 2 days for pain relief. Following recovery, the mice were given unrestricted freedom to move for 8 weeks. Mice were then euthanized using CO_2_ and the (experimental as well as control) knees were dissected and prepared for histological analysis.

### Histology and scoring

Hind limbs dissected from *Matn1*^*-/-*^ and *Matn1*^+/+^ mice at 8 weeks post-operative were fixed overnight in 4% paraformaldehyde in phosphate-buffered saline (pH 7.4), decalcified in 0.2M EDTA for 3 weeks, dehydrated in 70%, 95%, and 99.9% ethanol, cleared in xylene, and embedded in paraffin, and 6 μm sections were cut. Four sagittal sections were taken through the medial aspect of each specimen such that the articular surfaces of the tibia and femur were framed in the image. For Safranin-O/Fast Green staining, 5-μm paraffin-embedded sections of tibia from mice were counterstained with hematoxylin before being stained with 0.02% aqueous Fast Green for 4 minutes (followed by 3 dips in 1% acetic acid) and then 0.1% Safranin-O for 6 minutes. The slides were then dehydrated and mounted with crystal mount medium.

Eight sagittal sections were cut through the medial side of each specimen. All 8 sections from each specimen were considered separately and blindly by 2 investigators. Comparison of the *Matn1*^*-/-*^ and *Matn1*^*+/+*^ cartilage degradation was determined using a scoring system for the mouse knee developed by the Osteoarthritis Research Society International (OARSI) [24]. The femur and tibial articular surfaces on each section were scored on the 6-point OARSI scale. Sections in which there was no clear medial meniscus to help identify anatomic landmarks were discarded, leaving each specimen with 3 to 8 sections in which femur and tibial surfaces were clearly visualized. The OA scores for a given tibia and femur per specimen were defined as the maximum score for any of the sections. The maximum scores generated for each specimen were averaged together, producing four data sets: *Matn1*^*+/+*^ femur scores, *Matn1*^*+/+*^ tibial scores, *Matn1*^*-/-*^ femur scores, and *Matn1*^*-/-*^ tibial scores.

### Statistical analysis

Histology grading was analyzed using a Kruskale-Wallis test with Dunn’s post-analysis. Gene expression and mechanical testing was done using two-way analysis of variance (ANOVA) followed by t-test analysis. Statistical significance was accepted at P < 0.05 for all analyses.

## Results

### Gene expression analysis

**Fig 1:**
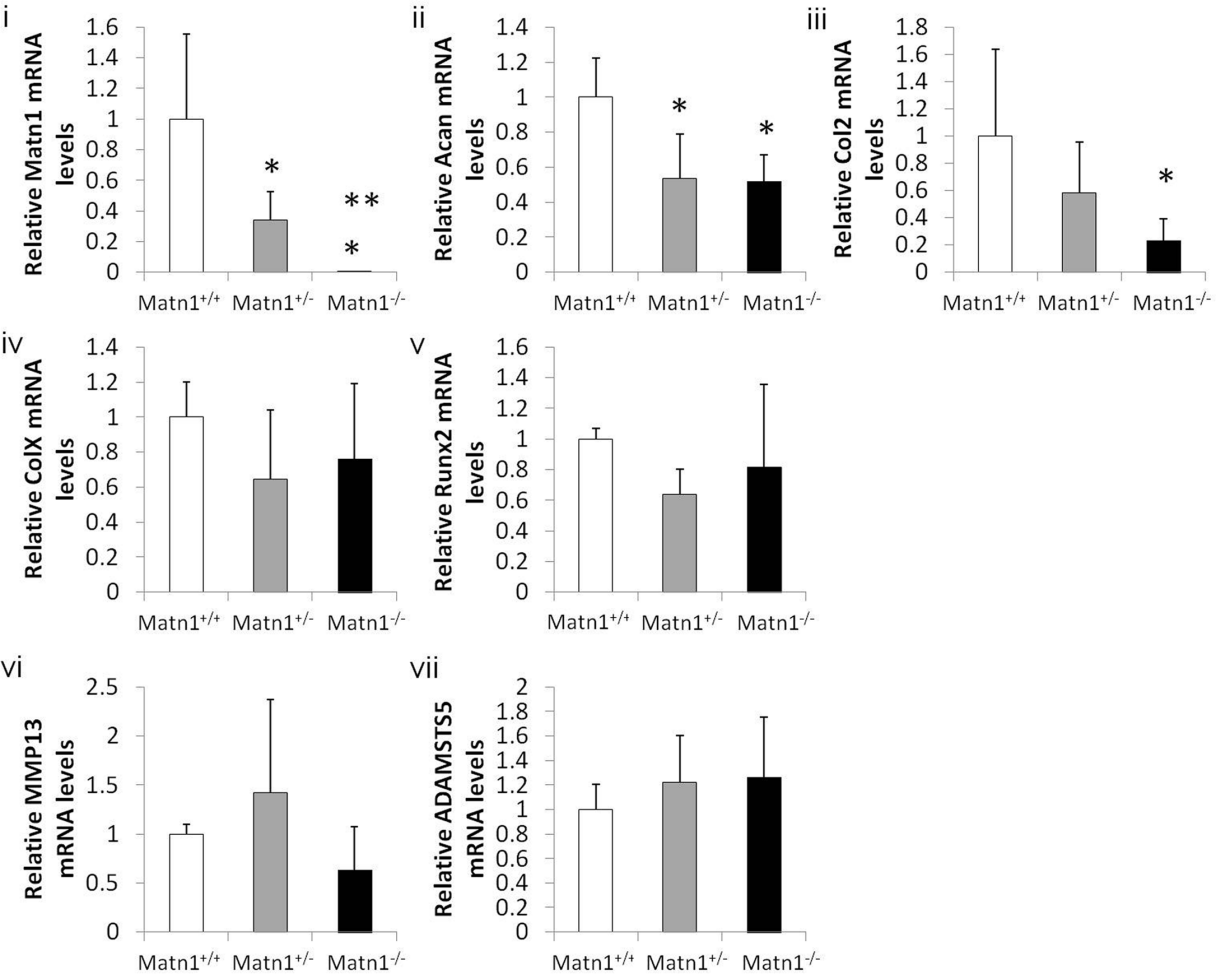
Gene expression of articular cartilage from *Matn1*^*-/-*^, *Matn1*^*+/-*^ and *Matn1*^*+/+*^ mice. Total RNA was isolated from femoral heads of 3 week-old *Matn1*^*+/+*^, *Matn1*^*-/-*^ and *Matn1*^*+/-*^ mice. Gene expression analysis was conducted by real time quantitative PCR (RT-qPCR). The cDNA of each sample was subjected to RT-qPCR using species-specific primer pairs for anabolic genes encoding i) *Matn1*, ii) aggrecan (*Acan*), iii) type II collagen (*Col2a1*); hypertrophic markers iv) type X collagen (Col X), v) runt-related transcription factor 2 (Runx2); and catabolic genes encoding vi) matrix metalloproteinases 13 (MMP13), and vii) a disintegrin and metalloproteinase with thrombospondin motifs 5 (ADAMTS5) Values are the mean ± SD. *p<0.05 compared to *Matn1*^*+/+*^, **p<0.05 compared to *Matn1*^*+/-*^. (n≥3)

To determine whether gene expression of articular cartilage was altered in *Matn1*^*-/-*^ mice, the mRNA levels of anabolic, catabolic, and hypertrophic markers were quantified via real time RT-PCR. Results confirmed the lack of *Matn1* mRNA transcripts in the femoral head cartilage of *Matn1*^*-/-*^ mice and reduced *Matn1* transcript levels in *Matn1*^*+/-*^ mice, relative to *Matn1*^*+/+*^ animals (Fig 1, i). Expression of anabolic genes, including cartilage ECM components (type II collagen and aggrecan), was significantly lower in the *Matn1*^*-/-*^ and *Matn1*^*+/-*^ mice compared with *Matn1*^*+/+*^ mice (Fig 1, ii and iii). *Matn1*^*-/-*^ animals exhibited 50% less *Acan* and 80% less *Col2a1* mRNA transcripts relative to *Matn1*^*+/+*^ animals. However, neither hypertrophic markers (*Col X* and *Runx2*) nor catabolic genes (*MMP13* and *ADAMTS5*) showed a statistically significant difference among *Matn1*^*-/-*^, *Matn1*^*+/-*^ and *Matn1*^*+/+*^ mice (Fig 1, v-vii). Thus, anabolic transcripts (*Acan* and *Col2a1)* levels were significantly reduced in *Matn1* deficient cartilage.

### Mechanical testing of articular cartilage

**Fig 2:**
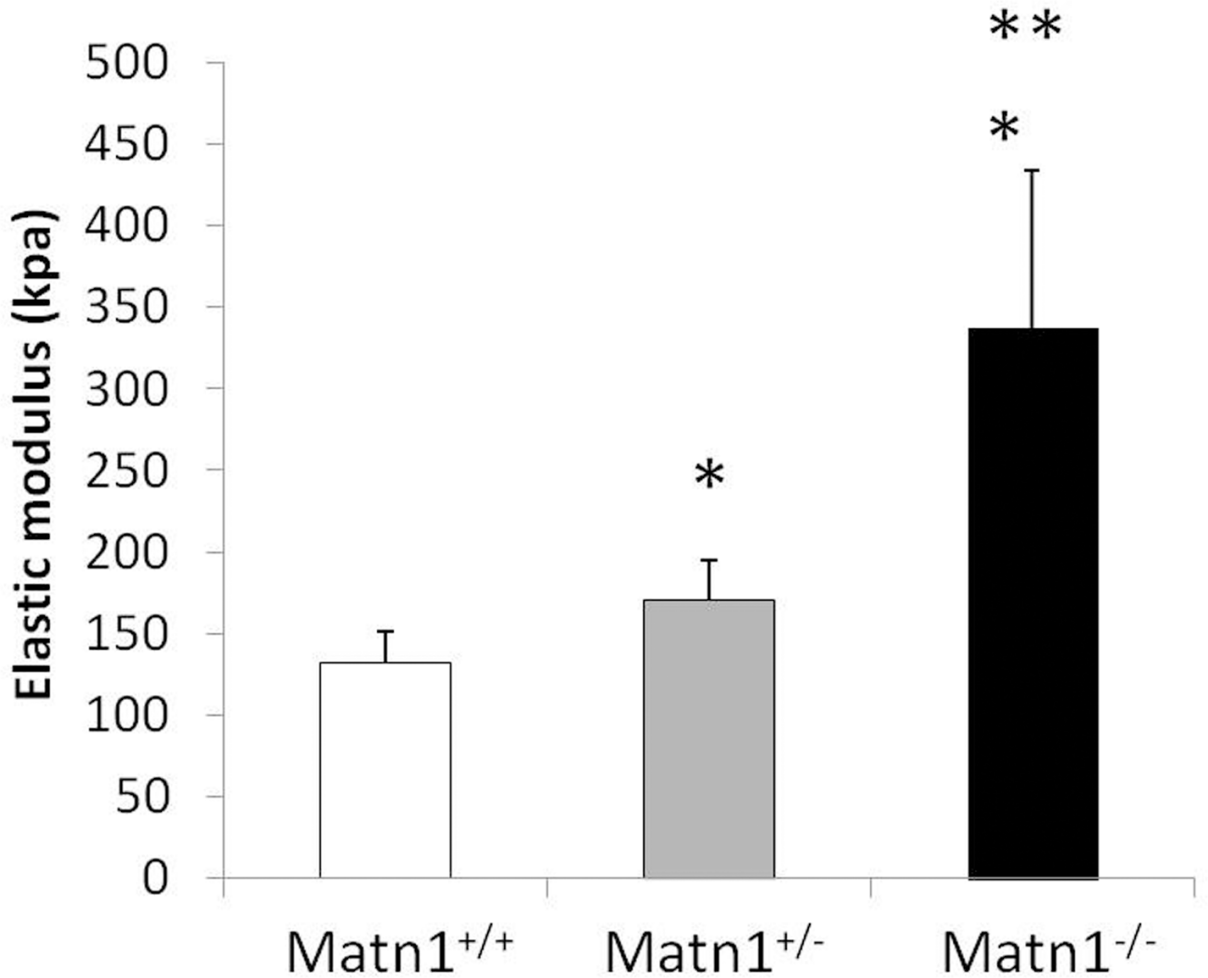
Quantification of elastic modulus by Atomic Force Microscopy (AFM) mechanical testing on articular cartilage surface of *Matn1*^*+/+*^, *Matn1*^*+/-*^ and *Matn1*^*-/-*^ mice. The femoral heads of 3 week-old mice were harvested and glued to a low profile 50 mm Petri dish to allow unobstructed access to the femoral head, and submerged in PBS to prevent drying of the tissue. Elastic moduli were quantitatively evaluated using an MFP-3D AFM. For each testing region, 100 data points were collected, resulting in n = 400–600 indentations for each genotype group included in the study. Indentation curves were fit using a modified Hertz model to extract an elastic modulus for the cartilage surface. Values are the mean ± SD. *p<0.05 compared to *Matn1*^*+/+*^, **p<0.05 compared to *Matn1*^*+/-*^. (n = 4–6)

To determine whether the mechanical properties of cartilage tissue were altered in *Matn1*^*-/-*^ mice, the elastic modulus of the surface of femoral head articular cartilage was quantified by atomic force microscopy (AFM). An average of 500 data points were collected from arrays of test sites at multiple locations areas on each specimen. The elastic modulus of *Matn1*^*-/-*^ mice was significantly higher than that of *Matn1*^*+/-*^ and *Matn1*^*+/+*^ mice, with *Matn1*^*-/-*^ cartilage exhibiting an elastic modulus (300 ± 70 kPa) double that of *Matn1*^*+/+*^ cartilage (150 ± 60 kPa) (Fig 2). Therefore, the mechanical property of cartilage matrix was altered in *Matn1*^*-/-*^ mice in comparison to *Matn1*^*+/+*^ mice.

### Transcriptome analysis of chondrocytes

**Fig 3:**
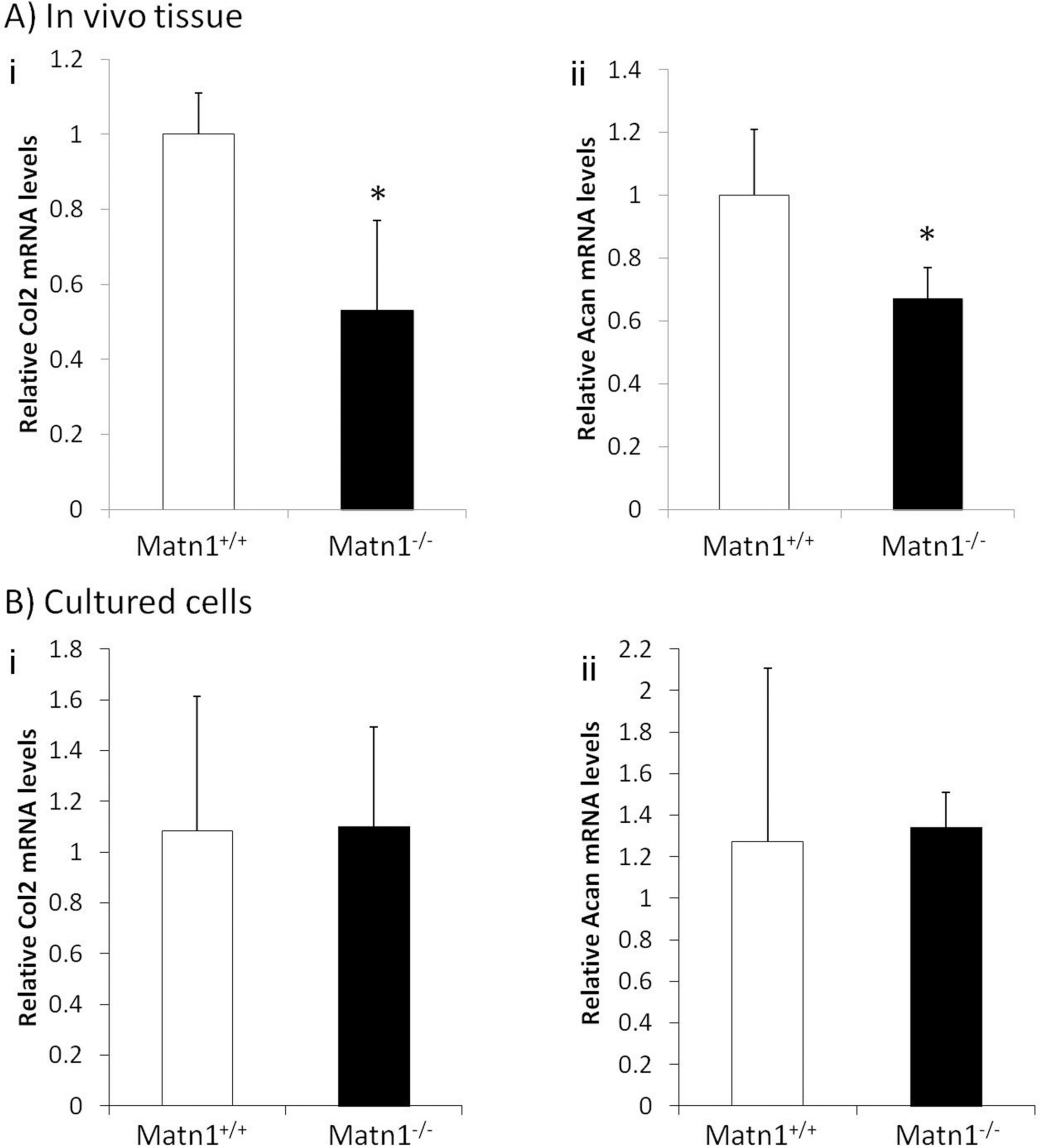
**Comparison of gene expression levels in A) cartilage of *Matn1***^***-/-***^ **and *Matn1***^***+/+***^ **mice, B) primary chondrocytes of *Matn1***^***-/-***^ **and *Matn1***^***+/+***^ **mice cultured under monolayer cell culture conditions.** Total RNA was isolated from A) rib cartilage and B) cultured primary chondrocytes of rib cartilage of 1 week old *Matn1*^*+/+*^ and *Matn1*^*+/+*^ mice. Gene expression analysis was conducted by real time quantitative PCR (RT-qPCR). The cDNA of each sample was subjected to RT-qPCR using for anabolic genes encoding i) type II collagen (*Col2a1*); ii) aggrecan (*Acan)*. Values are the mean ± SD. *p<0.05. (n = 3)

To determine whether the reduction of anabolic transcripts in *Matn1* deficient cartilage is consistent at different sites and ages, we quantified *Col2a1* and *Acan* mRNA levels in femoral head and rib cartilage from one week old mice. There is a significant reduction of *Col2a1* and *Acan* mRNA levels in both femoral head and rib cartilage (S1 Fig and Fig 3A). The reduction of anabolic transcripts (*Acan* and *Col2a1)* levels in *Matn1* deficient cartilage could be due to an internal transcription deficiency in the nucleus of *Matn1*^*-/-*^ chondrocytes. On the other hand, since *Matn1* is a peri-cellular and ECM protein, the lack of it may affect chondrocyte transcription through cell signaling from the extracellular environment. To distinguish these two possibilities, we isolated chondrocytes from mouse cartilage by digesting ECM and plated the cells in monolayer without external mechanical loading. Real-time PT-PCR analysis demonstrated that, although the anabolic transcripts (*Acan* and *Col2a1)* levels were reduced in *Matn1* deficient cartilage (Fig 3A), there was no significant difference in *Col2a1* and *Acan* mRNA levels between *Matn1*^*+/+*^ and *Matn1*^*-/-*^ chondrocytes isolated from cartilage (Fig 3B). This indicates that there is no internal deficiency of transcription of these anabolic transcripts in *Matn1*^*-/-*^ chondrocytes in comparison to *Matn1*^*+/+*^ chondrocytes. To further determine whether there is significant difference in the overall expression of transcriptome between *Matn1*^*+/+*^ and *Matn1*^*-/-*^ chondrocytes, microarray analysis of 28,206 probe sets (gene transcripts) was performed using mRNA isolated from *Matn1*^*+/+*^ and *Matn1*^*-/-*^ chondrocytes in monolayer culture. Principal Components Analysis indicated that there was no significant difference in the overall gene expression profiles between *Matn1*^*+/+*^ and *Matn1*^*-/-*^ chondrocytes. Therefore, the basal levels of gene transcription, except *Matn1*, were similar between *Matn1*^*+/+*^ and *Matn1*^*-/-*^ chondrocytes.

### Mechanical response of chondrocytes

**Fig 4:**
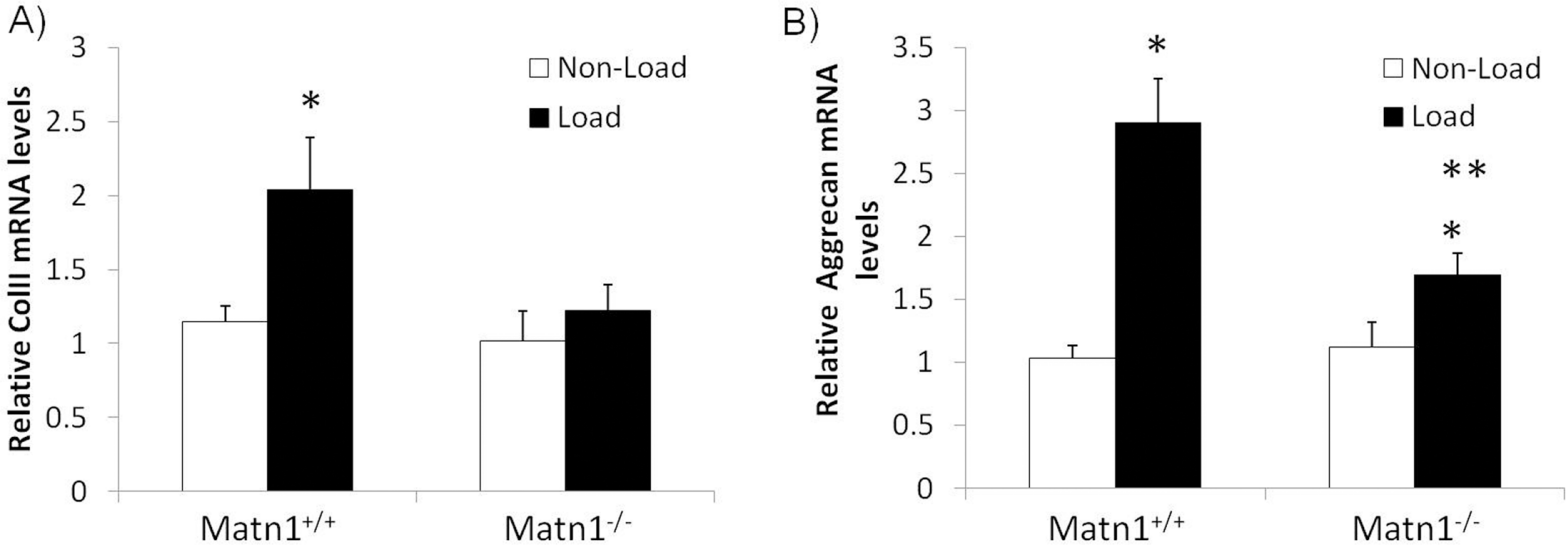
Comparison of gene expression levels in three-dimensional cell cultures under Load and Non-load conditions. Chondrocytes were cultured in 3D collagen scaffoldings, which were mechanically loaded to induce 5% elongation at 60 cycles/min, 15 min/h by a computer-controlled device. After 24 hours, total RNA was isolated from chondrocytes in 3D culture. Gene expression analysis was conducted by real time quantitative PCR (RT-qPCR). The cDNA of each sample was subjected to RT-qPCR using for anabolic genes encoding A) type II collagen (*Col2a1*); B) aggrecan (*Acan)* under both Load and Non-Load conditions. Values are the mean ± SD. *p<0.05 compared to non-load controls. **p<0.05 compared to *Matn1*^*+/+*^ samples with mechanical loading. (n = 3)

To test the possibility that the lack of *Matn1* in ECM may affect chondrocyte transcription through mechanical signaling from the extracellular environment, we cultured *Matn1*^*-/-*^ and *Matn1*^*+/+*^ chondrocytes in a three dimensional collagen sponge subjected to cyclic loading of matrix scaffold (1 Hz, 5% matrix deformation, 15 min/hr). The levels of anabolic transcripts including *Col2a1* and *Acan* mRNAs were quantified using real-time RT-PCR. In response to cyclic loading, *Col2a1* and *Acan* mRNA expression levels were significantly increased in *Matn1*^*+/+*^ mouse chondrocytes (Fig 4, *Matn1*^*+/+*^). In contrast, mechanical stimulation of *Col2a1* mRNA was abolished in *Matn1* deficient chondrocytes (Fig 4A, *Matn1*^*-/-*^). In addition, mechanical stimulation of *Acan* mRNA levels was significantly decreased in *Matn1* deficient chondrocytes in comparison to *Matn1*^*+/+*^ chondrocytes (Fig 4B, *Matn1*^*-/-*^). Furthermore, the difference of anabolic transcripts expression levels between *Matn1*^*+/+*^ and *Matn1*^*-/-*^ chondrocytes only exist under mechanical loading (Fig 4, compare Load samples between *Matn1*^*+/+*^ and *Matn1*^*-/-*^), and there is no statistically significant difference of these transcripts under non-load conditions (Fig 4, compare Non-Load samples between *Matn1*^*+/+*^ and *Matn1*^*-/-*^). Thus, cyclic loading induced anabolic transcripts in *Matn1*^*+/+*^ chondrocytes and such induction was abolished in *Matn1*^*-/-*^ chondrocytes.

### Cartilage degeneration in response to altered mechanical environment

**Fig 5:**
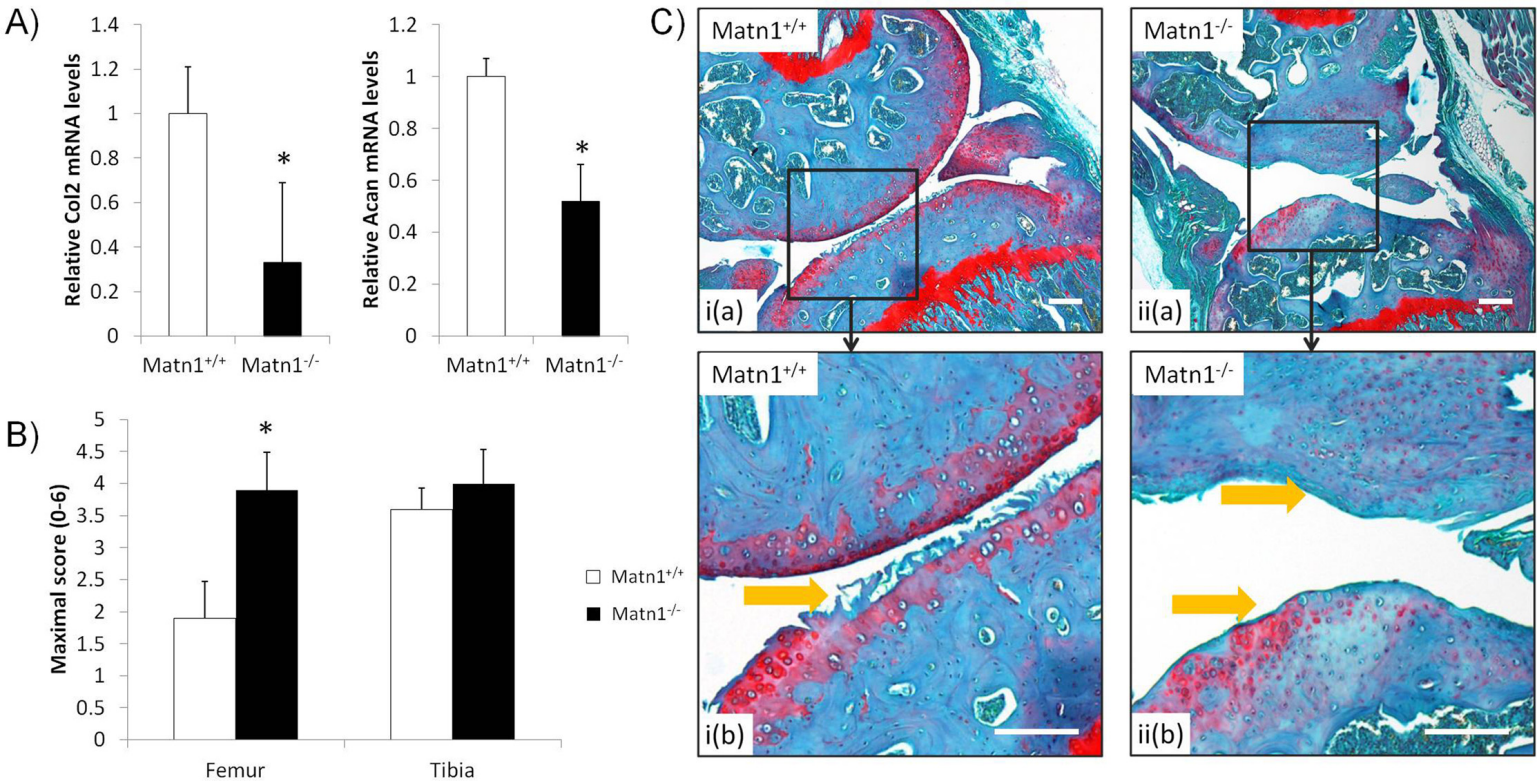
Characterization of knee cartilage degeneration. Destabilization of the medial meniscus (DMM) was performed on the right knees of 2 month-old male *Matn1*^-/-^ (n = 11) and *Matn1*^+/+^ (n = 9) mice. A) mRNA levels of type II collagen (*Col2a1*) and aggrecan (*Acan)* in femur head cartilage of *Matn1*^*+/+*^ and *Matn1*^*-/-*^ mice before DMM surgery. B) Maximum OARSI scores of articular cartilage from femur and tibia knees of *Matn1*^*+/+*^ and *Matn1*^*-/-*^ mice two months after DMM. The femur and tibial articular surfaces on each section were scored on the 6-point OARSI scale. The OA scores for a given tibia and femur per specimen were defined as the maximum score for any of the sections. The maximum scores generated for each specimen were averaged together, producing four data sets: *Matn1*^*+/+*^ femur scores, *Matn1*^*+/+*^ tibial scores, *Matn1*^*-/-*^ femur scores, and *Matn1*^*-/-*^ tibial scores. Values are the mean ± SEM. *p<0.05 compared to *Matn1*^*+/+*^. (n≥9). C) Representative histology sections of knee cartilage two months after DMM procedure. 5-μm paraffin-embedded sections of mice knee were stained for Safranin-O/Fast Green and counterstained with hematoxylin. i) *Matn1*^*+/+*^ and ii) *Matn1*^*-/-*^ mice at lower (a) and higher (b) magnifications (Yellow arrows pointing to the lesions at the articular surface.), bar = 500nm,

Deficiency of mechanical activation of anabolic chondrocytes may affect cartilage degeneration in response to altered mechanical loading environment. To test this hypothesis, we altered mechanical loading of articular cartilage by destabilization of medial meniscus (DMM) in the mouse knee. In mouse knee articular cartilage, the mRNA levels of *Acan* and *Col2a1* were significantly reduced in *Matn1*^*-/-*^ mice (Fig 5A), similar to rib and femoral head cartilage. Histology analysis was performed following DMM procedure. Histological scoring indicated that the *Matn1*^*-/-*^ femur cartilage sustained a greater degree of erosion than the *Matn1*^*+/+*^ cartilage. The results showed that the average maximum OARSI score for *Matn1*^*-/-*^ femoral cartilage was 3.9 ± 1.5, significantly higher than *Matn1*^*+/+*^ (1.9 ± 1.5) (p<0.05), (Fig 5B, Femur). On the other hand, the *Matn1*^*+/+*^ tibial cartilage was more severely damaged by DMM procedure than femoral cartilage and there was no statistically significant increase of the OARSI score in *Matn1*^*-/-*^ tibial cartilage in comparison to *Matn1*^*+/+*^ (Fig 5B, Tibia).

In addition, the overall morphology of cartilage erosion patterns differed between *Matn1*^*-/-*^ and *Matn1*^*+/+*^ specimens (Fig 5C). While fissures and clefts of the superficial layer were more commonly seen in *Matn1*^*+/+*^ cartilage specimens (Fig 5C i), irregular and massive degenerative matrix changes predominated in *Matn1*^*-/-*^ specimens (Fig 5C ii). Loss of proteoglycan is evident in articular cartilage of the *Matn1*^*-/-*^ specimens, as indicated by the severe reduction of Safranin O staining in the matrix. Furthermore, the cartilage zones and surrounding matrix are disorganized, suggesting deficiency in ECM organization in *Matn1*^*-/-*^ articular cartilage.

## Discussion and Conclusions

The function of MATN1 *in vivo* was not known, since cartilage development was normal in *Matn1*^*-/-*^ mice [11]. However, previous studies have shown that the diameters of collagen fibrils in cartilage were increased [2,11] suggesting the content and/or mechanical properties of ECM in *Matn1*^*-/-*^ mouse cartilage may be altered. We hypothesize that Matn1 may play a role in protecting cartilage from degeneration and such a role may only manifest when cartilage is tested under altered mechanical environment. To test this hypothesis *in vivo*, we performed destabilization of medial meniscus (DMM) procedure in *Matn1*^*-/-*^ mice. This approach was utilized because Matn1 is expressed specifically in mature chondrocytes [1]. The cartilage tissue and differentiation stage specific expression of Matn1 ensures that any phenotypic changes are due to chondrocyte autonomous effects in cartilage, but not from effects of other tissues *in vivo*. The second important consideration is that the morphology of growth plate and articular cartilage is apparently normal in the *Matn1*^*-/-*^ mice [11]. This eliminates the alteration of cartilage morphology as a confounding variable for studying cartilage’s response to mechanical loading. Third, we have shown previously that the content of pericellular Matn1 is critical to optimal chondrocyte mechanotransduction *in vitro* [12]. We tested whether this conclusion also holds true *in vivo* in this study.

Alteration of mechanical loading to cartilage joint is performed by DMM procedure. It is selected for its relatively mild effect of mechanical damage on cartilage matrix and for its minimal activation of pro-inflammatory pathways in the joint [23]. Histology analysis indicates that, following DMM procedure, instead of typical fissures and clefts commonly seen in articular cartilage surface of *Matn1*^*+/+*^ mice, irregular and massive degenerative erosion predominated in *Matn1*^*-/-*^ mice. Notably, proteoglycan accumulation is diminished and zonal organization of chondrocytes is lacking in *Matn1*^*-/-*^ cartilage. This strongly suggests that the mechanism underlying degenerative changes in *Matn1*^*-/-*^ mice is more severe than that of the *Matn1*^*+/+*^ mice. Interestingly, such histological changes are only seen in *Matn1*^*-/-*^ mice following DMM but not in those mice without DMM. Thus, altered mechanical environment triggers manifestation of the new cartilage degenerative phenotype in *Matn1*^*-/-*^ mice. These results demonstrated, for the first time, the importance of MATN1 in cartilage homeostasis and its preventive role in OA progression. Such properties have only been revealed in Matn1 deficient mice under post-traumatic conditions.

Histology analysis suggests that Matn1 deficiency may alter the content of ECM in cartilage. Such alteration could be due to the reduction of synthesis and/or increased degradation of ECM. To test this, we quantified expression of key homeostatic genes expressed by chondrocytes. First, we analyzed the anabolic genes of major ECM components in cartilage, specifically, *Acan* and *Col2a1*. Both genes were down-regulated in *Matn1*^*-/-*^ mice. Chondrocytes from *Matn1*^*+/-*^ mice also showed a trend of lower expression of both genes indicating that the production of ECM was suppressed in *Matn1*^*-/-*^ mice. This is the first evidence to show that ECM production is affected by Matn1 deficiency in mammals. In support of this conclusion, a recent study has shown that collagen production and secretion is reduced when *Matn1* is knocked down in zebra fish [25]. We also quantified mRNA levels of *Col6a1* and *Decorin*, which interact with *Matn1*. However, they were not significantly different between the *Matn1*^*+/+*^ and *Matn1*^*-/-*^ mice (data not shown). Therefore, the effect of *Matn1* on matrix production may be gene specific.

Because cartilage degeneration can be caused not only by the decrease of anabolic gene expression, but also by activation of chondrocyte hypertrophy and increase of catabolic gene expression [26], we quantified the expression of hypertrophic markers and catabolic genes in cartilage. Neither the expression levels of hypertrophy markers Col X and Runx2 nor those of catabolic genes MMP13 and ADAMTS5 were significantly different among *Matn1*^*+/+*^, *Matn1*^*-/-*^ and *Matn1*^*+/-*^ mice. These findings strongly suggest that decrease of ECM production, but not stimulation of chondrocyte hypertrophy or matrix degradation was responsible for the more severe cartilage lesions observed in *Matn1*^*-/-*^ mice during PTOA.

Changes of ECM production may result in alteration of material properties of cartilage matrix. To test whether the mechanical property of Matn1 deficient cartilage was altered, we quantified the elastic modulus of mouse articular cartilage surface by AFM. Surprisingly, the elastic modulus of *Matn1*^*-/-*^ mice was more than double that of *Matn1*^*+/+*^ mice, indicating a much stiffer matrix in *Matn1*^*-/-*^ cartilage. There are at least two explanations as to why the stiffness is increased in *Matn1*^*-/-*^ cartilage. First, due to the lack of *Matn1* on the surface of collagen fibrils, the collagen fibrils aggregate to form dense collagenous structure [9–11]. Since type II collagen has a long half-life, the accumulated, dense collagen fibrils contribute to the increase of the elastic modulus despite the decrease of new collagen production in *Matn1*^*-/-*^ cartilage. On the other hand, the half-life of aggrecan is much shorter than that of collagens in the matrix. Since the decrease of proteoglycan content has been shown to increase cartilage stiffness [27], the decrease of proteoglycan production would significantly impact cartilage mechanical properties in Matn1 deficient mice.

Chondrocyte mechanotransduction could be affected by altered matrix property as well as by the lack of peri-cellular Matn1, which has previously been shown to be responsible for transducing matrix deformation signals to chondrocytes *in vitro* [12]. To test cellular response to mechanical loading, we cultured *Matn1*^*+/+*^ and *Matn1*^*-/-*^ mouse chondrocytes in 3D collagen scaffolding subjected to cyclic loading. In response to mechanical loading, the mRNA levels of *Col2a1* and *Acan* were significantly increased in *Matn1*^*+/+*^ chondrocytes. However, this mechanical stimulation is diminished in *Matn1*^*-/-*^ chondrocytes. Thus, *Matn1*^*-/-*^ chondrocytes are deficient in stimulating ECM production in response to mechanical strain. Interestingly, our results suggested that the synthetic defect of *Matn1*^*-/-*^ chondrocytes is not the cause of the ECM production deficiency. *Matn1*^*-/-*^ chondrocytes have the same *Col2a1* and *Acan* mRNA levels as the *Matn1*^*+/+*^ chondrocytes when cultured in monolayer and in 3D cultures without external loading. Furthermore, there is no significant difference in the overall expression profiles of transcriptome between *Matn1*^*+/+*^ and *Matn1*^*-/-*^ chondrocytes, as indicated by microarray analysis. This suggested that a major defect of *Matn1*^*-/-*^ chondrocytes is the lack of sensitivity to mechanical environment, rather than any deficiency in mRNA synthesis. This enables *Matn1*^*-/-*^ mice to be an ideal model for testing the role of mechanotransduction *in vivo*.

Although chondrocytes residing in mouse articular cartilage show a significant difference in anabolic gene expression (i.e., *Acan* and *Col2a1*) between *Matn1*^*+/+*^ and *Matn1*^*-/-*^ mice *in vivo*, such difference is lost when the same chondrocytes are isolated from the surrounding ECM and cultured *in vitro*. This difference in anabolic gene expression, however, can be restored by applying external cyclic loading *in vitro*. These observations suggest that articular cartilage is a mechanically active tissue *in vivo*, and that matrix molecules such as Matn1 is essential for chondrocytes to adapt to mechanical environment. Thus, when mechanical loading environment is altered, such as during PTOA, chondrocytes in *Matn1*^*+/+*^ mice are able to adapt to mechanical stress by increasing the synthesis of ECM molecules such as Col II, Acan, and matrilins [28,29], while chondrocytes in Matn1 deficient mice fail to adapt to mechanical stress. This failure results in a diminished ability of chondrocytes to maintain cartilage homeostasis, which eventually leads to more severe cartilage lesions during PTOA. One limitation of the study is that the mRNA levels by RT-PCR analysis have not been confirmed by protein analysis due to technical challenges. Such analysis at protein levels will be performed in future experiments.

**Fig 6:**
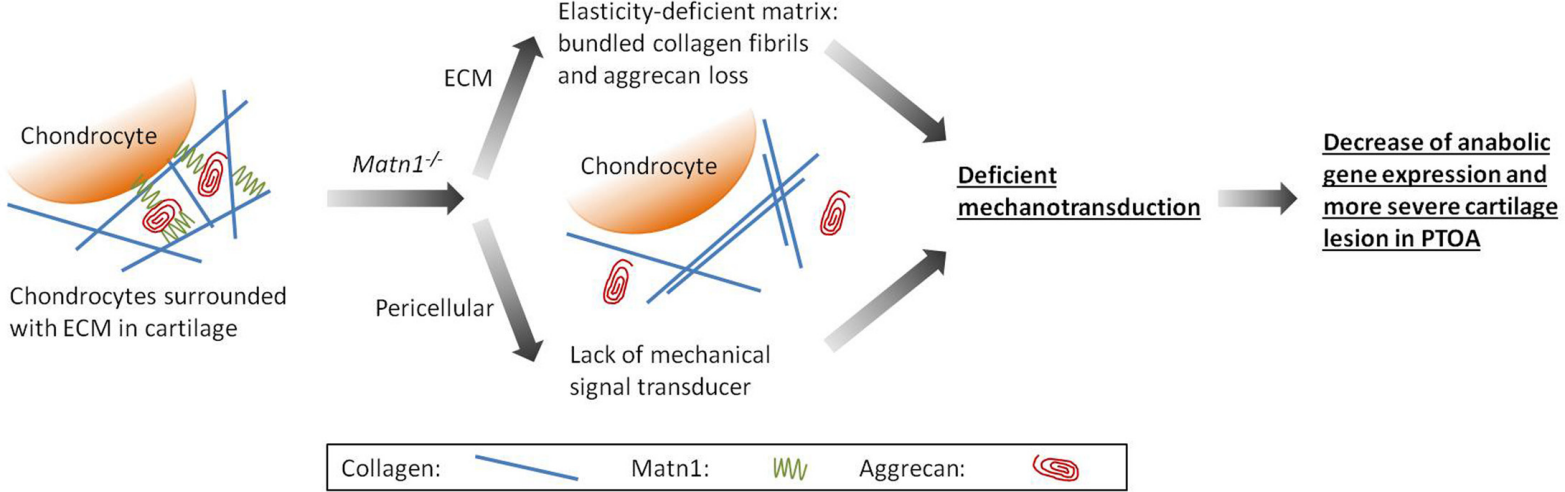
Schematic diagram illustrating a proposed mechanism of the role of Matn1 in mechanotransduction for adaptation to mechanical environment in cartilage. The lack of Matn1 results in aggregation of collagen bundles and decrease of aggrecan content. These changes lead to stiff matrix mechanical property, which, together with the absence of pericellular Matn1, diminish chondrocyte mechanotransduction ability.

In summary, our working model (Fig 6) is that mechanotransduction plays an important role for chondrocytes to adapt to altered mechanical environment during PTOA. This important role is revealed by the analysis of *Matn1*^*-/-*^ mice presented here. The lack of Matn1 results in aggregation of collagen bundles and decrease of aggrecan content. These changes lead to stiffer matrix mechanical property, which, together with the absence of pericellular Matn1, diminish chondrocyte mechanotransduction ability. Our data suggests that, in addition to the commonly recognized stimulation of catabolic genes, the failure of stimulation of anabolic genes by mechanical loading also contributes to pathogenesis of OA.

## Supporting Information

S1 Fig Gene expression levels of young articular cartilage from *Matn1*^*-/-*^ and *Matn1*^*+/+*^ mice. Total RNA was isolated from femoral head of 1 week-old *Matn1*^*+/+*^, *Matn1*^*-/-*^ mice. The cDNA of each sample was subjected to RT-qPCR using species-specific primer pairs for anabolic genes encoding aggrecan (*Acan*) and type II collagen (*Col2a1*). Values are the mean ± SD. *p<0.05 compared to *Matn1*^*+/+*^ (n≥3).
(TIF)
