# Supplementary figures and images for: Deficient Mechanical Activation of Anabolic Transcripts and Post-Traumatic Cartilage Degeneration in Matrilin-1 Knockout Mice
Source: PLoS One. 2016 Jun 7;11(6):e0156676. doi: 10.1371/journal.pone.0156676 (PMC4896629; doi:10.1371/journal.pone.0156676)

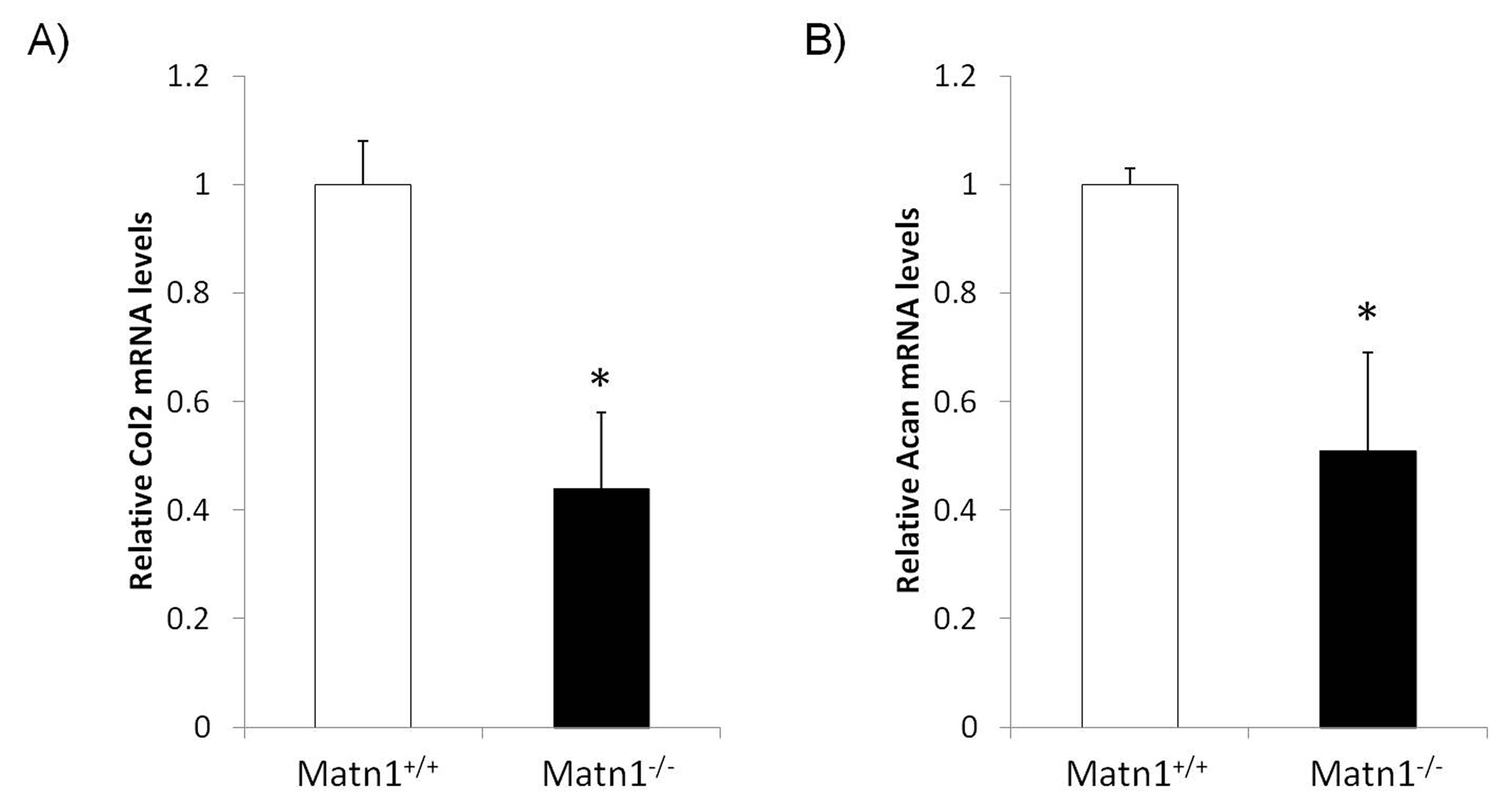

Supplement: S1 Fig — Total RNA was isolated from femoral head of 1 week-old Matn1+/+, Matn1-/- mice. The cDNA of each sample was subjected to RT-qPCR using species-specific primer pairs for anabolic genes encoding aggrecan (Acan) and type II collagen (Col2a1). Values are the mean ± SD. *p<0.05 compared to Matn1+/+ (n≥3). (TIF) [file pone.0156676.s001.tif]
